# Tomographic Evaluation of Alveolar Ridge Preservation Using Bone Substitutes and Collagen Membranes—A Retrospective Pilot Study

**DOI:** 10.3390/dj11030058

**Published:** 2023-02-22

**Authors:** Tegan S. Binkhorst, Andrew Tawse-Smith, Rayner Goh, Getulio R. Nogueira, Momen Atieh

**Affiliations:** 1 Sir John Walsh Research Institute, Faculty of Dentistry, University of Otago, Dunedin 9016, New Zealand; 2 Hamdan Bin Mohammed College of Dental Medicine, Mohammed Bin Rashid University of Medicine and Health Sciences, Dubai 505055, United Arab Emirates

**Keywords:** alveolar ridge preservation, bone substitutes, collagen membranes, retrospective study

## Abstract

Alveolar ridge preservation (ARP) reduces dimensional changes following tooth extraction. We evaluated the changes in alveolar ridge dimensions after ARP using bone substitutes and collagen membranes. Objectives included the tomographic evaluation of sites prior to extraction and six months after ARP and the assessment of the extent ARP preserved the ridge and reduced the need for additional augmentation at the time of implant placement. A total of 12 participants who underwent ARP in the Postgraduate Periodontics Clinic (Faculty of Dentistry) were included. Cone beam computed tomography images were used to retrospectively assess 17 sites prior to and six months after dental extraction. Alveolar ridge changes were recorded and analysed using reproducible reference points. The alveolar ridge height was measured at buccal and palatal/lingual aspects, whilst width was measured at crestal level, 2 mm, 4 mm and 6 mm below the crest. Statistically significant changes were found in alveolar ridge width at all four heights, with mean reduction differences ranging from 1.16 mm to 2.84 mm. Likewise, significant changes in the palatal/lingual alveolar ridge height (1.28 mm) were observed. However, changes of 0.79 mm in buccal alveolar ridge height were not significant (*p* = 0.077). Although ARP reduced dimensional changes following a tooth extraction, some degree of alveolar ridge collapse could not be avoided. The amount of resorption on the buccal aspect of the ridge was less compared to the palatal/lingual after ARP. This indicated that the use of bone substitutes and collagen membranes was effective in reducing changes in the buccal alveolar ridge height.

## 1. Introduction

Tooth loss is the end stage of oral disease, often due to extensive decay, periodontal disease and, less commonly, trauma or poor alignment. Tooth loss leads to a decrease in one’s quality of life, affecting masticatory ability, communication and socialisation. Restorative and/or prosthetic rehabilitation must be carefully planned due to the consequences for the oral tissues post-extraction. Dental implants are a common procedure to replace lost teeth, either by supporting a fixed or removable prosthesis [1].

Following tooth extraction, substantial alterations to the width and height of the alveolar ridge occur [1,2,3]. In the interest of achieving long-term esthetically acceptable implant support, rehabilitation efforts have been made to maintain and or decrease the inevitable modelling and remodelling process of the alveolar ridge following tooth extraction [3]. The bundle bone contains investing periodontal ligament fibres, which act to anchor teeth into the jaw. As it is a tooth-dependent structure, following tooth extraction, bundle bone loses its function and resorbs over time [1,4]. The residual ridge is reduced most rapidly in the first six months, but the bone resorption activity continues throughout life, albeit at a slower rate, resulting in the resorption of a large amount of jaw structure. In 2005, Araújo and Lindhe studied mongrel dogs and found an absence of bundle bone in specimens four to eight weeks after premolar extraction. This resulted in a significant reduction in the alveolar ridge height and width, particularly on the buccal aspect. Human clinical studies have shown up to 50% ridge width reduction following premolar and molar extractions, with two-thirds of the dimensional changes occurring in the first three months [2,4,5]. Furthermore, the wall thickness of the buccal bone influences the degree to which bone loss occurs. Thinner buccal bone walls of less than 1 mm consist mainly of bundle bone, resulting in extensive resorption after tooth extraction [4,6].

Alveolar ridge preservation (ARP) is synonymous with terms such as socket grafting, socket preservation, socket augmentation and ridge preservation [7,8,9,10]. It is a concept first suggested in 1994 that is a regenerative intervention on sites which require delayed implant placement [11]. Regardless of technique, dimensional changes after extraction cannot be completely avoided. However, ARP can be used to reduce the extent of these changes [4]. ARP predictably reduces vertical and horizontal ridge resorption, allowing for prosthetically driven delayed implant placement [7,10]. ARP involves the use of grafting materials to fill the extraction socket and is often covered with a membrane [7,12]. This procedure is commonly used prior to delayed implant placement [7,8,9]. Grafting materials have specific biological, chemical and mechanical properties, making them suitable for use in humans. The main hallmark of these biomaterials, which are biocompatibility, degradability, adhesion, osteogenic properties, induction properties and low immunogenicity, allow them to be utilised in regenerative processes [13]. ARP often involves resorbable or non-resorbable barrier membranes which maintain the space for bone infiltration. Non-resorbable membranes, such as cellulose acetate fibres (Millipore), expanded polytetrafluoroethylene (e-PFTE/Teflon), or titanium built into e-PFTE, require a second surgery to be removed prior to implant placement. However, these often result in a greater amount of bone fill and have good marginal tissue response [7]. Resorbable membranes, such as polyglycolide synthetic copolymers, collagen and calcium sulphate, show good soft tissue healing and do not require a second surgery [7,10].

The mechanism of action of bone substitutes in ARP falls under three main categories: osteogenesis, osteoinduction or osteoconduction. Osteogenesis results in the formation of new bone from surviving osteoprogenitor cells within the graft [14,15]. Osteoconduction is where the grafting material acts as a scaffold allowing the interdigitation of osteoprogenitor and capillary cells. This is a common property seen in most bone grafting materials. Osteoinduction is where bone growth is stimulated via the differentiation of osteoblasts from mesenchymal cells [7]. Grafting materials used in ARP include autografts, allografts, xenografts, alloplasts, growth factors and collagen sponges. Autografts are harvested from the patient via the use of a bone trap or hand instruments. Extra-oral (from the iliac crest) or intra-oral (from the mandibular ramus, symphysis or maxillary tuberosity) harvesting techniques are not common due to morbidity [7]. Autografts are ideal as no immune response is invoked [16]. Allografts are from the same species, with decreased morbidity to the patient, as a second surgical site is not required. Mineralised frozen or freeze-dried bone (FDBA) allografts are primarily osteoconductive in nature, whereas demineralised frozen or demineralised freeze-dried bone (DFDBA) may also have osteoinductive properties. Studies have shown that there is no difference in changes in ridge dimension following ARP between these two types of allografts [7,10,17]. Xenografts come from different species, such as deproteinised bovine bone material (DBBM), which is mainly osteoconductive in nature [7,18]. Studies suggest that there is no difference in ridge preservation following ARP using a membrane alone or a membrane in conjunction with DBBM [7,19,20]. There are a variety of alloplasts (synthetic materials) which are osteoconductive and inert [7]. While there is a multitude of materials and techniques used for ARP, the current gold standard is still the use of autologous bone (autografts), due to the optimal osteogenetic, osteoinductive and osteoconductive properties of the grafting material [14,15].

The aim of this study was to evaluate the dimensional changes of the alveolar ridge following the placement of bone substitutes and collagen membranes in a university setting. This was achieved by tomographic evaluation of sites prior to extraction and six months after ARP to determine the extent of ridge preservation following the use of such materials. This was achieved by analysing changes in height and width after these procedures. Hypotheses were that ridge resorption is expected following extraction and ARP and that the buccal bone will undergo more extensive resorption.

This pilot study provides information to clinicians about the outcome of ARP by postgraduate students in a university setting. Less favourable outcomes have been demonstrated in implant procedures by less experienced clinicians, and it is interesting to observe the differences in ARP between training clinicians in this study and other reported data from experienced clinicians [21].

## 2. Materials and Methods

This retrospective pilot study took place within the Postgraduate Periodontic Clinic at the University of Otago Faculty of Dentistry in New Zealand. This involved the use of clinical notes and associated cone-beam computed tomography (CBCT) images of participants who received ARP treatment. Each participant underwent CBCT imaging prior to and six months after tooth extraction and ARP. All participants were seen by Periodontic postgraduate students within the Faculty of Dentistry for ARP procedures as part of their periodontal and dental implantology treatment. This study falls under the ‘Minimal Risk Health Research—Audit and Audit related studies’ category and thus has ethical approval from both the University of Otago Human Ethics Committee (departmental approval from the Sir John Walsh Research Institute, approval code HD22/019), as well as from the Ngāi Tahu Research Consultation Committee.

Inclusion criteria consisted of adult participants who had CBCT images prior to tooth extraction and six months after the ARP procedure; complete and adequate clinical documentation, which included the type of bone graft and barrier membrane used, and the type of adjunctive antibiotic (if used). Exclusion criteria included participants without CBCT images; or insufficient information on the grafting materials and membrane used.

In order to find suitable participants, patient records from the Faculty of Dentistry at the University of Otago were filtered by treatment codes used in Titanium (the clinical software used in the Faculty of Dentistry)—including codes 026 (CBCT image), 236 (guided tissue regeneration) and 243 (osseous graft). This ensured that selected patients had received treatment involving ARP procedures, as well as preoperative and postoperative CBCT imaging. These patients underwent these procedures between 2018 and 2021. A total of 12 participants (with 17 total sites) were selected for this study. The data collected from these participants included demographic and clinical information. Demographic information included participants’ age, sex, tooth site(s), smoking status and diabetic status. Clinical information included clinical notes pertaining to the materials used for ARP (graft and membrane), adjunctive antibiotic (if applicable) and CBCT images at baseline and six months after extraction.

CBCT images were analysed using GALILEOS Implant software (Dentsply Sirona). Firstly, the right arch design (shape) and apico-coronal position were selected by setting the panoramic curve for the correct arch. From the tangential view of the site, a slice was taken where apices of adjacent teeth were visible, with the greatest length of the root.

**Figure 1:**
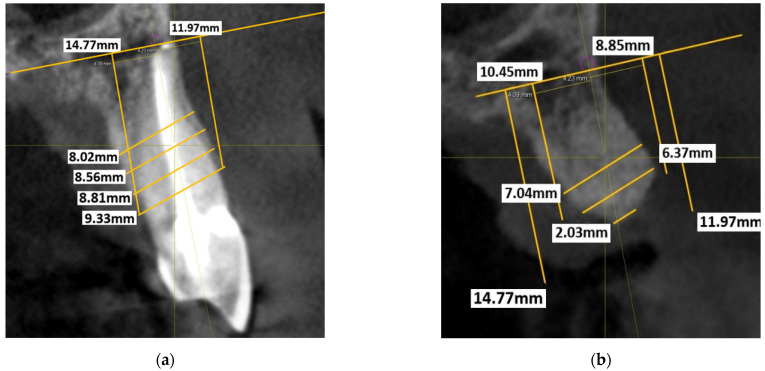
(**a**) Preoperative measurements of left maxillary canine site; (**b**) Postoperative measurements of left maxillary canine site. Note the loss of vertical height causing the thickness measurements to start at 2 mm below the height of the original crests (outer parallel lines).

A reference line was then traced intersecting the highest point of these apices. The length was recorded, as well as any landmarks, such as tracing a line between the maxillary sinus and anterior nasal spine. The axial view was also checked to confirm that the slice was centred in both the buccolingual and mesiodistal direction of the site. The previously mentioned reference line was used to orientate between the preoperative and postoperative CBCTs, to standardise the measurements recorded. Along this previously mentioned reference line, a ‘nerve point’ was traced with a 1 mm diameter. In the cross-sectional view, this nerve point acts as the reference point for the topographical measurements. The cross-sectional view was orientated using the vertical reference line through the long axis of the tooth. By using the 90° angle tool, a line was drawn intersecting the bottom of the nerve point, perpendicular to the vertical reference line. From this apical trace line, two perpendicular lines were drawn, buccal and lingual/palatal, parallel to the vertical reference line at the highest point of the crests. This noted the distance from the vertical reference line to the buccal and lingual/palatal lines for standardisation. The length of these lines indicated the height of the respective crests. These vertical lines were then joined together with another line (crest height line). Lines were drawn parallel to the crest height line at 2 mm, 4 mm and 6 mm intervals. These lines were drawn from the most external aspect of the ridge on both the buccal and lingual/palatal aspects. The length of each respective line indicated the thickness of the ridge. These measurements are shown in Figure 1a.

These height and thickness measurements were repeated on the second CBCT (Figure 1b) using the previously mentioned reference lines six months after tooth extraction. Postoperative ridge height was taken from the highest part of the alveolar crest on both the buccal and lingual/palatal aspects. The height and thickness of the ridge were measured and compared with the preoperative measurements.

All data (demographic and clinical measurements) was recorded in Excel spreadsheets and analysed using Statistical Package for Social Sciences (SPSS) Version 28 for Windows. Intra-examiner agreement was assessed using the intra-class correlation coefficient (ICC). Measures of skewness and kurtosis and Shapiro–Wilk tests were used to assess the normality of data. A paired two-tailed *t*-test was used to identify differences in alveolar ridge dimensions between the two points in time (before extraction and six months after ARP). Statistical significance is indicated with a *p*-value less than 0.05.

## 3. Results

In Table 1, the demographic and clinical information of the participants is outlined. The participants were aged between 20 and 79 years and were categorised into age groups (e.g., 20–29 years old, 70–79 years old). The mean age of the participants was 55 years, with a standard deviation of 17 years. All participants were systemically healthy except for one participant who was pre-diabetic. More male participants were included compared to females. Most participants were non-smokers or former smokers, with only one participant who was a current smoker who reported occasional smoking habits. A greater proportion of sites in the maxilla were measured, with an equal number of anterior (incisors and canines) and posterior (premolar and molars) sites included from this arch. Three different bone substitutes were used: Bio-Oss^®^ and Endobon^®^ (xenografts) and Puros^®^ (allograft). Most sites had Bio-Oss^®^ placed as the bone substitute. Two barrier membranes were used: Bio-Gide^®^ and OsseoGuard^®^. All participants except two had antibiotics prescribed after the ARP procedure, with a range of antibiotics used (shown in Table 1 above).

**Figure 2:**
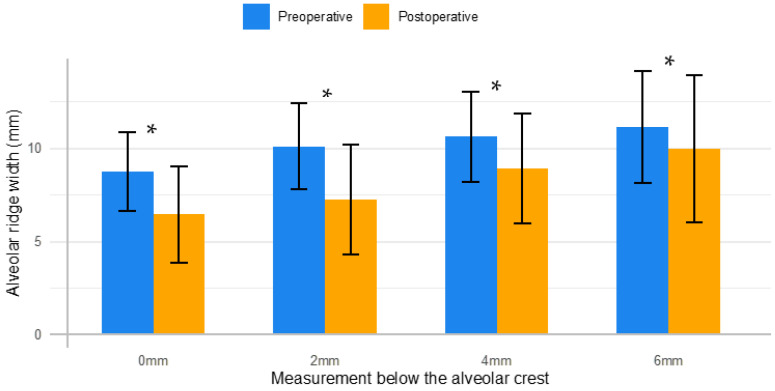
Alveolar ridge width measurements. Mean width of the alveolar ridge preoperatively (blue) and postoperatively (orange) is shown at crestal level (0 mm), 2 mm, 4 mm and 6 mm below the crest. * Statistically significant mean difference (*p* < 0.05).

**Figure 3:**
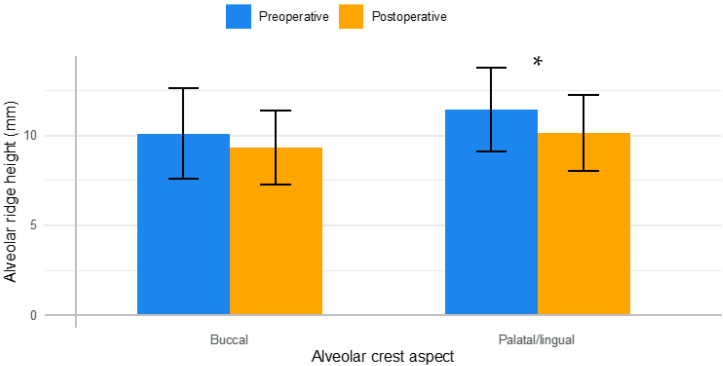
Alveolar ridge height measurements. Mean preoperative (blue) and postoperative (orange) crestal height measurements are shown for both the buccal and palatal/lingual aspects of the alveolar crest. * Statistically significant mean difference (*p* < 0.05).

The mean alveolar ridge widths and vertical heights, as well as their associated mean differences between preoperative and postoperative measurements, are outlined in Figure 2 and Figure 3.

As the normality assumptions were satisfied, parametric tests were applied. There was a mean reduction in the thickness of the alveolar ridge width for all four measurements below the height of the crest (0 mm, 2 mm, 4 mm and 6 mm). The mean differences were 2.30 ± 1.35 mm, 2.84 ± 2.48 mm, 1.70 ± 1.65 mm and 1.16 ± 1.28 mm at 0 mm, 2 mm, 4 mm and 6 mm below crestal level, respectively. These mean differences for the changes in the alveolar ridge width were statistically significant (*p* < 0.05).

For alveolar ridge height, a statistically significant mean difference of 1.28 ± 1.46 mm was found between preoperative and postoperative measurements pertaining to the palatal/lingual aspect of the alveolar crest, as seen in Table 2. However, a marginally significant mean difference of 0.79 ± 1.72 mm was found for changes in the buccal aspect of the alveolar crest (*p* = 0.077). The ICC ranged between 0.97 and 0.99 indicating excellent intra-rater reliability.

## 4. Discussion

As hypothesised, the results of this study showed dimensional changes occurred in both the width and height of the alveolar bone. Although ARP reduces the extent of alveolar ridge resorption, it cannot prevent the physiological bone remodelling that occurs post-extraction [4]. However, studies have shown that the use of grafting materials (xenografts or allografts) and barrier membranes resulted in better outcomes compared to unassisted healing, with a mean effect of 2.1 mm for height (mid-buccal) and 1.9 mm for width (buccolingual) [4,22].

For alveolar ridge width, we found the mean reduction differences between preoperative and postoperative measurements ranged from 1.16 mm–2.84 mm, with the greatest amount of resorption occurring in the crestal area rather than the base of the socket. The mean reduction differences in the alveolar ridge height were found to be 1.28 mm for the palatal/lingual and 0.79 mm for the buccal aspect of the alveolar crest. This is in accordance with other results in current literature, such as a systematic review from Horváh et al. (2013) which investigated dimensional changes after ARP [23]. This systematic review found horizontal width changes varying from −1.0 mm to −3.5 ± 2.7 mm and mean mid-buccal height changes between +1.3 ± 1.9 mm and −0.5 ± 1.1 mm. Lim et al. (2019) researched molar sites grafted with Bio-Oss^®^ and Bio-Gide^®^ and found horizontal changes of −1.02 ± 0.88 mm, −0.31 ± 1.51 mm and 0.04 ± 1.29 mm at levels 1 mm, 3 mm and 5 mm below the crest, respectively [24]. This study also found vertical changes of −0.58 ± 0.53 mm in the buccal crest height and −0.12 ± 1.10 mm in the lingual crest height. Conversely, in molar sites grafted with Bio-Oss^®^ alone, they found changes of −2.49 ± 3.34 mm, −1.17 ± 1.33 mm and −0.59 ± 0.98 mm in the alveolar ridge width at 1, 3 and 5 mm below crestal level, respectively, −1.06 ± 1.57 mm in the buccal crest height and −0.33 ± 0.38 mm in the lingual crest height.

From the 17 sites retrospectively assessed in this study, nine have had an implant placed at the Faculty of Dentistry to date. From these nine sites, five required minor grafting at the time of implant placement to further enhance the phenotype of the sites. Four of these sites received autografts collected during the implant osteotomy, whilst one received a xenograft. Due to the sample size within this study, and the fact that not all sites have received an implant at this stage, it is difficult to determine whether ARP reduced the need for additional augmentation. However, one could hypothesise that these sites may have undergone substantial dimensional changes without ARP and, therefore, not be suitable for implant placement.

Although marginally significant, the height of the buccal ridge appeared to be more conserved after ARP (0.79 ± 1.72 mm) compared to the palatal/lingual aspect (1.28 ± 1.46 mm). This indicates a favourable outcome in preserving the alveolar ridge height. This is particularly interesting as the buccal ridge tends to resorb further following a tooth extraction, due to the higher proportion of bundle bone present during healing, compared to the lingual/palatal aspect. The bundle bone resorbs as it is a tooth-dependent structure, causing a significant reduction in the height and width of the buccal wall [1,4]. Furthermore, sufficient buccal bone height (and thickness) is required to ensure that gingival margins have long-term stability around both implants and adjacent teeth [25].

Chappuis et al. (2013) also found that the thickness of the buccal bone influences the degree to which resorption occurs. An average of 7.5 mm in buccal bone height was lost after 8 weeks of healing. However, sites with a thicker buccal wall phenotype (greater than 1 mm) only experienced an average of 1.1 mm of vertical bone loss. When there was sound neighbouring dentition present, these extensive dimensional changes occurred mainly in the central area of alveolar socket walls (i.e., mid-buccal sites [4,6]. Therefore, buccal wall phenotypes could be included in subsequent research to investigate whether these phenotypes influence the level of resorption that occurs on the buccal aspect following ARP procedures.

As this study was retrospective, a small sample size of participants was included due to a lack of postoperative CBCTs prior to implant placement. Hence, this is considered a pilot study, and further research is needed in this area. If a greater pool of participants were available to provide more statistical power, comparisons could be made between demographics and the efficacy of ARP. For example, it would be interesting to investigate whether there was a difference in ridge preservation between the grafting materials; Bio-Oss^®^ (xenograft), Puros^®^ (allograft) and Endobon^®^ (xenograft). A recent meta-analysis from Natto et al. (2017) found that xenografts with a barrier membrane were the most efficacious in alveolar ridge width preservation, whilst FDBA (allograft) was the most efficacious in ridge height preservation. However, the results from this study were not statistically significant. Regardless, it would be interesting for future research to investigate whether the outcomes of ARP follow this potential trend [26]. As mentioned in the table above (Table 1), 10 out of the 12 patients received antibiotics after ARP as they are commonly used in adjunct to ARP procedures [27]. Antibiotic prophylaxis in implant placement procedures is used to prevent infections and may reduce the risk of early implant loss [28]. Further research is needed to investigate whether antibiotics influence the healing outcomes following ARP. Finally, most participants were non-smokers or former smokers, with only one participant recording occasional smoking habits. This is a positive to note, as smoking negatively affects healing after tooth extraction and has been found to increase the resorption of the alveolar ridge width [23,29]. This was also demonstrated in our study, as the participant who occasionally smoked cigarettes had 3.41 mm of horizontal bone loss at the crest compared to the average value of 2.30 ± 1.35 mm. However, the change in the alveolar ridge height was similar to that of other participants in this study. Again, this is limited by the small sample size and should be evaluated further in a larger-scale study.

Great care was used to standardise preoperative and postoperative measurements. However, some differences may have occurred as this research was retrospective, and no stent could be used to ensure identical reference points were used for measurements. Furthermore, the GALILEOS Implant software license held by the University of Otago Faculty of Dentistry does not include slice numbers present when viewing CBCT images, increasing the difficulty of standardisation. Dimensional changes after ARP vary considerably based on the presenting ridge morphology. In the present study, some participants presented with thick buccal bone at the crestal aspect and fenestrations were present at the buccal aspect of the ridge below the crest, which may result in fewer changes observed at the crestal area compared to 2 mm below the crest. This can be explained by dimensional changes being influenced by variabilities in the ridge morphology. Future research in this area could involve a prospective cohort study design, with the use of both stents and slice numbers, to ensure maximum standardisation between CBCT images. Most of the patients were treated with Bio-Oss^®^ grafting material and Bio-Gide^®^ collagen membranes, which limited the generalisation of the results to other regenerative materials. Other limitations of this study included the limited sample size and a limited number of mandibular sites, as there were only three posterior mandibular sites and no anterior mandibular sites, as well as the wide range of patient age groups. Due to the retrospective nature of the study, some confounding factors that may have affected the results have been excluded. Patient compliance with the home care instructions and antibiotic regimen was not noted down by the clinicians and was therefore not reported in this study. The ARP procedures were also carried out by postgraduate students at various stages of the training program. However, no major complications were noted for the patients included in this study.

As mentioned prior, substantial dimensional changes in the alveolar ridge occurred following tooth extraction [1,2]. This poses complications for adequate tooth replacement, both aesthetically and functionally. For functional implant placement to occur, a maintained alveolar ridge is required [3]. Even though no ARP technique will completely prevent dimensional changes from occurring, the extent of these changes can be reduced [4]. Therefore, research on this subject is important, as knowledge of ARP techniques and their efficacy could help drive functional and aesthetic implant placement and reduce the likelihood of complications and prosthetic failure [4,30].

## 5. Conclusions

As hypothesised, alveolar ridge resorption still occurred following extraction with ARP. Therefore, some degree of alveolar ridge collapse cannot be avoided. Loss of width of the alveolar crest was found at all four measurement points below the height of the crest (0 mm, 2 mm, 4 mm and 6 mm). Interestingly, the palatal/lingual aspect of the alveolar crest underwent greater resorption compared to the buccal bone, which refuted our hypothesis. However, this indicates a favourable outcome for preserving the alveolar ridge height after extraction, as this is important for prosthetically-driven implant placement.

## Figures and Tables

**Table 1:** Table outlining participant demographic and clinical information.

**Participants**	12					
Age (years, mean ± SD)	55.83 ± 17.71					
Systemic Conditions	None		Diabetes		No Med Hx	
	10		1 (pre-diabetic)		1	
Sex	Male			Female		
	8			4		
Smoking	Never		Former	Smoker	No Med Hx	
	6		4	1	1	
Sites	Total number of sites					
	17					
	Maxillae			Mandible		
	Anterior		Posterior	Anterior	Posterior	
	7		7	0	3	
Bone substitute/Graft	Per Site					
	Bio-Oss^®^		Puros^®^		Endobon^®^	
	Anterior	Posterior	Anterior	Posterior	Anterior	Posterior
	5	6	2	2	0	2
	11		4		2	
Membrane	Per Site					
	Bio-Gide^®^			OsseoGuard^®^		
	Anterior		Posterior	Anterior	Posterior	
	5		6	2	4	
	11			6		
Adjunctive antibiotics	Per Patient					
	Amoxicillin 500 mg		Amoxicillin 500 mg + Metronidazole 400 mg	Amoxicillin 500 mg + Metronidazole 200 mg	Clindamycin 300 mg	Clindamycin 150 mg
	6		1	1	1	1

**Table 2:** Preoperative and Postoperative mean changes in alveolar ridge height and width.

	Mean (SD)	Mean Difference (SD)	95% CI	*p*-Value
Changes in alveolar ridge height				
Buccal (preoperative) Buccal (postoperative)	10.09 (2.51) 9.31 (2.05)	0.79 (1.72)	−0.96, 1.68	0.077
Palatal/lingual (preoperative) Palatal/lingual (postoperative)	11.42 (2.32) 10.14 (2.10)	1.28 (1.46)	0.53, 2.02	0.002
Changes in alveolar ridge width				
Crest (preoperative) Crest (postoperative)	8.77 (2.11) 6.47 (2.59)	2.30 (1.35)	1.39, 3.21	<0.001
Two millimetres (preoperative) Two millimetres (postoperative)	10.13 (2.29) 7.28 (2.96)	2.84 (2.48)	1.52, 4.16	<0.001
Four millimetres (preoperative) Four millimetres (postoperative)	10.64 (2.40) 8.93 (2.95)	1.70 (1.65)	0.85, 2.55	<0.001
Six millimetres (preoperative) Six millimetres (postoperative)	11.15 (3.00) 9.99 (3.59)	1.16 (1.28)	0.50, 1.81	0.002

## Data Availability

Data is available on request due to ethical restrictions.
